# Injuries of the Kidney

**Published:** 1914-06

**Authors:** Frank K. Boland

**Affiliations:** Atlanta, Ga.; Professor of Surgery, Atlanta Medical College


### Journal-Record of Medicine

Successor to Atlanta Medical and Surgical Journal, Established 1855
and Southern Medical Record, Established 1870

**OWNED BY THE ATLANTA MEDICAL JOURNAL COMPANY**

PUBLISHED MONTHLY

*Official Organ Fulton County Medical Society, State Examining Board, Presbyterian Hospital, Atlanta, Birmingham and Atlantic Railroad Surgeons' Association, Chattahoochee Valley Medical and Surgical Association, Etc.*

EDGAR BALLENGER, M. D., Editor
BERNARD WOLFF, M. D., Supervising Editor
A. W. STIRLING, M. D., C. M., D. P. H.; J. S. HURT, B. Ph., M.D.
GEO. M. NILES, M. D., W. J. LOVE, M. D., (Ala.); Associate Editors
R. R. DALY, M. D., Assiciate Editor
E. W. ALLEN, Business Manager

**COLLABORATORS**

DR. W. F. WESTMORLAND, General Surgery
F. W. McRAE, M. D., Abdominal Surgery
H. F. HARRIS, M. D., Pathology and Bacteriology
E. B. BLOCK, M. D., Diseases of the Nervous System
MICHAEL HOKE, M. D., Orthopedic Surgery
CYRUS W. STRICKLER, M. D., Legal Medicine and Medical Legislation
E. C. DAVIS, A. B., M. D., Obstetrics
E. G. JONES, A. B., M. D., Gynecology
R. T. DORSEY, JR., B. S., M. D., Medicine
L. M. GAINES, A. B., M. D., Internal Medicine
GEO. C. MIZELL, M. D., Diseases of the Stomach and Intestines
L. B. CLARKE, M. D., Pediatrics
EDGAR PAULIN, M. D., Opsonic Medicine
THEODORE TOEPEL, M. D., Mechano Therapy
A. W. STIRLING, M. D., Etc., Diseases of the Eye, Ear, Nose and Throat
BERNARD WOLFF, M. D., Diseases of the Skin
E. G. BALLENGER, M. D., Diseases of the Genito-Urinary Organs

VOL. LXI. ATLANTA, GA., JUNE, 1914. NO. 3

### INJURIES OF THE KIDNEY.

BY FRANK K. BOLAND, M. D.

Professor of Surgery, Atlanta Medical College.

On account of the comparative rarity of this condition, at first sight this paper might not appear to be one which would evoke general interest in this meeting. I offer two excuses for having made such a choice: first, because I have had five cases in the past eighteen months, for which reason such a paper need not be written altogether with scissors; and, second, because in this day of automobiles, airships, and increasing travel by all means, in spite of so-called safety appliances, the medical profession is being called upon more and more to treat serious injuries of the human body.

No doubt, in our student days, and even in our later reading, such headings in our text-books as "Injuries of the Kidney," "Rupture of the Spleen," etc., failed to attract our attention, and we hurried over the paragraph with the feeling that these were things we would never see, so why waste time on them? Such a conclusion was in the main correct, yet all of us have seen or will see, a few such rare cases, and when we do, we may wish that we had studied their symptomatology and treatment more diligently, because sometimes the outcome of such cases depends upon the promptness with which they are handled.

So, I trust a discussion of injuries of the kidney will not be amiss this afternoon. Statistics bear me out in the importance of the organ I have selected, showing that in 40% of subcutaneous injury of abdominal viscera, the kidney is the one affected. Additional interest is given the kidney over wounds of other internal organs in our ability to detect the lesion through hematuria, which occurs in 80% of cases. We have no such reliable guide in the diagnosis of injuries of other viscera.

At the outset injuries of the kidney may be classed under two distinct heads: (a) subcutaneous, or subparietal injury, without open wound, corresponding to simple fracture; and (b) injury with open wound, corresponding to compound fracture. Strange as it may seem to the lay mind, at least, the first variety is far more common. Open wounds, due usually to stabs and bullets, are exceedingly rare, especially in civil practice. Since they occupy but a small part of this discussion, they may be disposed of first.

Up to fiive years ago, according to Johnson, in "Surgical Diagnosis," less than 100 cases of stab-wound of the kidney had been reported, and not a single case was reported in the

Civil War. In the gun-shot wounds of war generally injury of the kidney forms .12% of the total.

Two cases of open wound of the kidney have fallen to my lot, one due to a long-bladed pocket knife, and the other to a bullet. The first patient was a white male, 35 years of age, with a stab-wound of the left loin. He was seen at the hospital immediately after the injury, and his general condition was good. Though he had lost considerable blood from the wound, it had not affected his pulse. His left rectus muscle was somewhat rigid, and fearing an intraperitoneal injury, he was anesthetized and the abdomen opened along the semilunaris. Nothing was seen except a subperitoneal hemorrhage of the anterior surface of the colon, and the wound was closed. Upon enlarging the stab-wound the twelfth rib was found cut in two, the kidney incised in two places, and the posterior wall of the colon lacerated, though the lumen was not opened. One wound, at the lower pole of the kidney, was made by the knife, while apparently the other wound, at the upper pole, was made by the sharp edge of the cut rib. The kidney was sutured, and the rest of the wound packed. Recovery was rapid and complete.

The case of a gun-shot wound in a young negro man was interesting in that the bullet entered the right side and went through the left kidney, without injuring any other organ. This case teaches the lesson that all persons with injuries which could in any way be conceived to involve the kidney should be catheterized promptly. Such a procedure is so simple, and usually gives most valuable information. I had already learned the lesson, but the hospital interne in charge of this case had not, and because he did not see how the bullet could have struck the kidney, had not troubled to order an immediate examination of the urine. An X-ray might have suggested kidney injury, but as the patient was brought in during the night, the Roentgenologist could not be obtained for several hours. The bullet had entered the right loin just below the last rib, about five inches from the median line, and we thought we could feel it deep under the skin on the left side, the same distance from the median line, four or five inches higher up.

Miscroscopic urinary examination showed only a slight amount of blood, and though the negro's condition was excellent, nephrotomy was done, not to control to first great danger of kidney injury, hemorrhage, but to control the second, sepsis, by drainage. The kidney was found shot squarely in two, and was drained. The bullet was removed from the tenth interspace, and the patient made an uneventful recovery.

Subcutaneous kidney injury, without external wound, is a remarkably interesting study. Francis S. Watson, of Boston, read an interesting paper on this subject in 1903, and it is from him that most of my figures are taken. At that time he had collected 660 cases. Of course, a great many cases occur which never find their way into the literature. No doctors are more guilty of thus sleeping over their opportunities than we of the South. Crawford W. Long started it when he failed to make public his great discovery, and we are following in his footsteps in this regard. If the medical profession of the South expects to take its place in the nation, it must write, write, write, and report to the medical world the work it is doing.

These wounds of the kidney occur usually from falls or from being run over, but Watson reports eleven cases due to muscular action alone. Thus, one man ruptured his kidney in lifting a sick woman across a bed. Hematuria persisted for three weeks, but the patient made a good recovery without operation. Another man ruptured his kidney, with fatal result, by the muscular effort made in delivering a blow at an opponent with whom he was boxing. But the most curious case of all is one reported by Voit of a woman whose kidney was ruptured by her husband's grasping her about the waist while waltzing. An abscess developed in this kidney, and the patient

In 20 cases of Watson's series, blows or falls upon the front of the abdomen are stated to have been the cause of laceration of the kidney, and in all but two of them this was the sole result of the accident. Why should the kidney be more liable to rupture after non-penetrating wounds than other organs?

Kuster attempted to answer this question by some experiments with empty kidneys and with kidneys full of fluid which he threw upon the floor. He found that only superficial lacerations were thus produced on the flaccid organs, while deep lacerations affected the full organs, from which he deducted the theory that subcutaneous rupture of the kidney is due to hydraulic pressure acting through full vessels and pelvis, and causing the organ to burst along lines for the most part radiating from the hilus toward the point of maximum impact of the lower ribs, the opposing resistance being supplied by the vertebral column.

This is a reasonable explanation of the frequency of subcutaneous kidney rupture as compared with that of other organs, and in my cases in which I operated upon the kidney, I found that rupture had taken place exactly along these lines, that is, at right angles to the long axis of the organ, radiating from the pelvis to the cortex. C. L. Gibson, in the Amer. Jour. Med. Sc., May, 1912, reported four cases in children in which the same thing took place.

To take up symptoms and diagnosis, the nature of the accident may or may not be considered, since we have seen that the kidney may be wounded from the most trivial causes. Pain, tenderness, tumor and hematuria are the most important signs. Shock is an uncertain symptom. It is usually present in some degree, but may be entirely absent, or very much delayed, as in a case of mine. Pain may be severe from the first, or only become so as the capsule becomes distended with blood, causing pressure upon nerves. The pain often radiates down the ureter to the bladder. Extreme tenderness over the kidney is usually present, but if not, the reflex spasm of the lumbar muscles on the affected side may be conspicuous. Renal colic, from obstruction by blood-clot, is significant. A tumor will form behind the peritoneum, unless that membrane is torn and allows the blood the escape into the cavity. In this case we will have abdominal distention, and signs of progressive anemia. Usually, if the effusion is considerable, the patient will lie with the thigh on that side flexed to relieve the tension of the muscles of the back and of the abdominal wall.

Blood in the urine is the most constant and, of course, the most characteristic sign of renal injury. While slight hematuria may not be taken as conclusive proof of demonstrable injury of the organ, if the quantity of blood be at all large it is presumptive evidence of a lesion of greater or less extent, and if tumor in the loin is present as well, the diagnosis is cinched. Hematuria may be slight or absent. If only the capsule of the kidney is torn, blood will not appear. Or in very severe injuries, where the ureter is torn across, or becomes stopped with blood-clot, the quantity of blood may be small, microscopic or absent. If the ureter is plugged, hematuria may be delayed for hours or days, or may be intermittent. Usually it is seen in the first passage of urine. Blood-clot in the ureter may cause renal colic, or in the bladder may cause great pain and constant desire to urinate. Blood-clot may stop the urethra and cause retention of urine and formation of a tumor above the pubes, necessitating washing out he bladder or even cystotomy.

We may be called upon to decide whether the blood comes from the kidney or bladder. Usually in hemorrhage from the kidney the blood is evenly mixed, while from the bladder the last urine is bloodiest. This may suggest the use of the cystoscope, and we may use this instrument later to determine by ureteral catheterization whether both kidneys are present, in case it is decided to perform nephrectomy. The cystoscope is often very valuable aid, but sometimes, even in the hands of experts, it fails to come up to our expectations.

Sepsis is the greatest danger after hemorrhage. It occurred in 68 of 486 of Watson's series, and caused almost an equal number of deaths as hemorrhage. Where the renal artery or vein is torn, death is almost certain to follow unless operative relief is offered quickly. The vein was found torn in 14 of Watson's cases, and the artery once. Next to complications, which may, of course, include anything, anuria is the most common cause of death. When of short duration, and recovery takes places, suppression of urine is to be referred to reflex inhibitory action upon the other kidney, because of injury to its mate, the latter's function being temporarily suspened by traumatism. If of longer duration than 36 or 48 hours, anuria is generally of grave significance.

In 4 of Watson's 660 cases only one kidney was present. This is much higher than the proportion usually given for this anomaly, which is about one case of absent kidney in every 2,000 persons. Watson does not state what operation, if any, was performed in these four cases.

Peritonitis is an infrequent complication. Tuffier has demonstrated by experiments on animals that no urine flows from the surfaces of lacerated renal wounds, and that in order to have urinary extravasation under such circumstances, the renal pelvis, or one of the calyses, must stand in communication with the renal surface through the wound. Also, the same experimenter has shown that the introduction of urine into the peritoneal cavity, provided it is made gradually once, or even repeatedly, if only intervals of sufficient length are made between the different introductions, does not cause peritonitis, whereas, if the flow is continuous, the contrary is the case. Another experiment demonstrates that the urine which flows from the divided ureter into the peritoneal cavity in dogs does not cause peritonitis and death for four or five weeks; also, that the peritoneum quickly protects itself from the encroachments of obnoxious fluids by the formation of adhesions.

The proper treatment of these cases often puts our surgical judgment to the test. The mildest cases, of course, if we know they are mild, require only expectant treatment, ice over the loin, light diet, and rest in bed until the symptoms clear up. Conservative measures might also be in order where both kidneys were injured, or where the complication of other viscera contraindicated operation. But in the presence of increasing symptoms, and indeed in the majority of all cases where the kidney is injured, or reasonably supposed to be, I believe that operation is the safest plan. It is much better to operate unnecessarily, than to lose a patient by failure or by delay in operating. Sometimes a positive diagnosis of kidney injury cannot be made except by exploration.

Watson's figures show a mortality of 27% in cases treated expectantly, 7% in cases treated by operation other than nephrectomy, and 25% in cases treated by nephrectomy. It is difficult to compare results in the two methods of operative treatment because manifestly the cases treated by removal of the kidney must have been worse than those treated by suture or packing. But the fact that any kind of operative treatment gives a lower mortality than the so-called expectant plan is most significant.

Of course, the lumbar route of reaching the organ is to be preferred unless there are complicating conditions in the abdomen to be treated, when laparotomy should be done. Often lumbar incision will be necessary for handling the kidney after the peritoneal work is done. Save the kidney, if possible, or save part of it. Sutures and packing can usually control hemorrhage. Where great trauma has been done, however, nephrectomy is often the operation of choice. Hemorrhage and sepsis are then most surely forestalled, while a conservative course is not always so safe. If we have not demonstrated the presence of two kidneys before performing nephrectomy, we must remember the frequency with which one is absent.

Two points I would emphasize in operations on the kidney. First, remember that the last thoracic, iliohypogastric, and ilioinguinal nerves lie between the quadratus muscle and the kidney. These nerves should be recognized when they come into the field of operation, and any or all of them may, and be carefully pulled aside. If it becomes necessary to divide one of them, the severed ends ought to be caught up in a suture, and once more united upon closing the wound. This is an important precaution, taught by Edebohls. I have seen failure to attend to this point lead to an annoying and persistent pain in the thigh and the parts supplied by these nerves after the operation. The second point is to always tie such important vessels as the renal artery and vein with silk or linen, never with catgut. I am familiar with a case where a catgut ligature slipped and caused the death of the patient the night after the operation.

Of the three cases of subparietal injury which I report, in the first the renal wound was but an incident in the accident, since the other lesions overshadowed it and quickly killed the patient. The two other cases are typical examples of this kind of injury; nephrectomy was done in both, and both patients recovered.

Case 1.—In this case the renal injury was one of the least of the patient's troubles, and the actual extent of it was not demonstrated. The patient was riding in an automobile when the tongue of a two-horse wagon struck him a violent blow just over the liver, then glanced off. There was considerable skin contusion, though no penetration, and the patient, a white man of 29, was profoundly shocked for 2 or 3 hours. When I saw him some twelve hours after the accident, his general condition was surprisingly good, pulse, respiration and temperature being normal. There were some abdominal pain and tenderness, and rigidity of the right rectus. He had not voided urine, and catheterization showed considerable blood in the urine, which he contiuued to pass, though in decreasing quantity, for the remaining 48 hours that he lived. Laparotomy was done through the right rectus, and the abdomen found full of blood clots, which came from a large rent in the posterior surface of the liver. Excepting a small tear in the mesocolon no other demonstrable wound was present. The kidney injury evidently was small, and probably consisted in an internal rupture transmitted from the blow on the anterior abdominal wall. The wound in the liver could not be stitched, and was packed, and the abdominal cavity drained. The ninth and tenth ribs were fractured. The patient died 48 hours after the operation. He did not develop peritonitis and had no more hemorrhage. I thought his death was due to shock of the sympathetic nervous system.

Case 2.—Eleven-year-old boy, who was thrown from a farm wagon and struck his right loin on a rock on the ground. He was stunned for a moment, then arose and plowed two furrows before he fell from weakness. There were no external signs of violence, but considerable pain and tenderness over the loin, and the attending physician at first thought the twelfth rib was broken, and applied adhesive plaster. A few hours later, when the boy passed blood in his urine, and began to have temperature of 100 to 101, with a very weak pulse, he was brought to the hospital with the diagnosis of ruptured kidney. By this time he was passing considerable blood, and swelling in the loin was marked. There was no renal colic as has been reported in such cases from blood clots in the pelvis or ureter. Urination was not difficult or painful. Lumbar incision was made, and the fatty capsule was found full of blood clots, torn almost in two at the junction of the lower and middle thirds, at right angles to the long axis, and deeply torn in the same direction at the junction of upper and middle thirds. The kidney substance was very friable, and as stitches did not hold well, a nephrectomy was done. As the ligatures on the renal vessels did not seem particularly secure, and the patient's condition was bad, a procedure was adopted which I have utilized on other occasions. A strong hysterectomy clamp was left on the pedicle, with the handle protruding through the wound, and removed in three days. A cigarette drain was left in for a few days. The patient made an uneventful recovery, and six months after the operation was reported to be in good health. The amount of urine secreted arose to normal soon after the operation. About ten ounces of normal saline was injected intravenously while the patient was on the table.

Case 3.—Elevator boy, 16 years old, who was caught between the floor of his car and the top of the door when the car was suddenly driven upward by an accident. His left thigh was dislocated into the obturator foramen, and his left leg broken in two places, one being a compound fracture. The dislocation was reduced at once. He presented a picture of profound shock from hemorrhage, complained of great pain in the region of the left kidney, and catheterization showed almost pure blood. Signs of external violence over the kidney were wanting. Lumbar incision showed large blood clots, and a kidney torn exactly in the same lines as in Case 2, only both lacerations were complete. The viscus was literally in thirds. Only the untorn capsule on the inner border held them together. Suturing seemed out of the question in such a desperate case, and the kidney was removed as rapidly as possible. Venous saline infusion was also used during this operation. Recovery seemed complete when the patient left the hospital two months later.

I draw three conclusions from my experience with these cases:

First, that such injuries are on the increase, and demand our careful study.

Second, that the diagnosis usually is not difficult.

Third, that operative treatment usually is best.

